# Interleukin-18 as a diagnostic marker of adult-onset Still’s disease in older patients: a case report and review of the literature

**DOI:** 10.1186/s13256-018-1735-7

**Published:** 2018-07-10

**Authors:** Daisuke Usuda, Yoshiki Furumura, Kento Takeshima, Ryusho Sangen, Yasuhiro Kawai, Yuji Kasamaki, Yoshitsugu Iinuma, Tsugiyasu Kanda

**Affiliations:** 1 Department of Infectious Diseases, Kanazawa Medical University, 1-1 Daigaku, Uchinada-machi, Ishikawa-ken, Japan; 2 Department of Community Medicine, Kanazawa Medical University Himi Municipal Hospital, 1130 Kurakawa, Himi-shi, Toyama-ken, Japan

**Keywords:** Interleukin-18, Adult-onset Still’s disease, Diagnostic marker, Elderly patients, Older patients

## Abstract

**Background:**

Adult onset Still’s disease is a systemic auto-inflammatory condition of unknown etiology characterized by intermittent spiking high fever, an evanescent salmon-pink or erythematous maculopapular skin rash, arthralgia or arthritis, and leukocytosis. Recently, a high level of interleukin-18 has been reported as a new characteristic marker. On the other hand no reports have been published on high interleukin-18 as a marker in older patients. We report a case of adult onset Still’s disease in an older patient successfully treated with steroids in which interleukin-18 was a useful marker of disease activity.

**Case presentation:**

A 66-year-old Asian woman presented to our hospital with fever and arthralgia. We diagnosed adult onset Still’s disease based on Yamaguchi criteria and a history of a high spiking fever, salmon-colored rash, and bilateral pain to shoulders, knees, and wrists. In this case, a high serum level of interleukin-18 was a diagnostic parameter. Administration of 40 mg of prednisolone followed by subcutaneous administration of 200 mg cyclosporine daily resulted in a dramatic resolution of our patient’s febrile episodes 2 months after admission. Prednisolone was tapered to 5 mg/day every 2 weeks and cyclosporine 200 mg/day was continued. Her serum interleukin-18 level was prominently decreased, and she was discharged 3 months after treatment.

**Conclusions:**

Serum interleukin-18 level may be a good diagnostic biomarker to monitor adult onset Still’s disease activity in older patients, measuring levels in both the acute and convalescent phases.

## Background

Adult onset Still’s disease (AOSD) is a systemic auto-inflammatory condition of unknown etiology, characterized by intermittent spiking high fever, an evanescent salmon-pink or erythematous maculopapular skin rash, arthralgia or arthritis, and leukocytosis [1]. High serum ferritin levels, elevated erythrocyte sedimentation rate, high C-reactive protein levels, and an absence of antinuclear antibody (ANA) and rheumatoid factor are common laboratory findings [2]. A high level of interleukin (IL)-18 has recently been reported as a new characteristic marker; however, no such report focusing on older patients has been published [3, 4].

## Case presentation

**Fig. 1:**
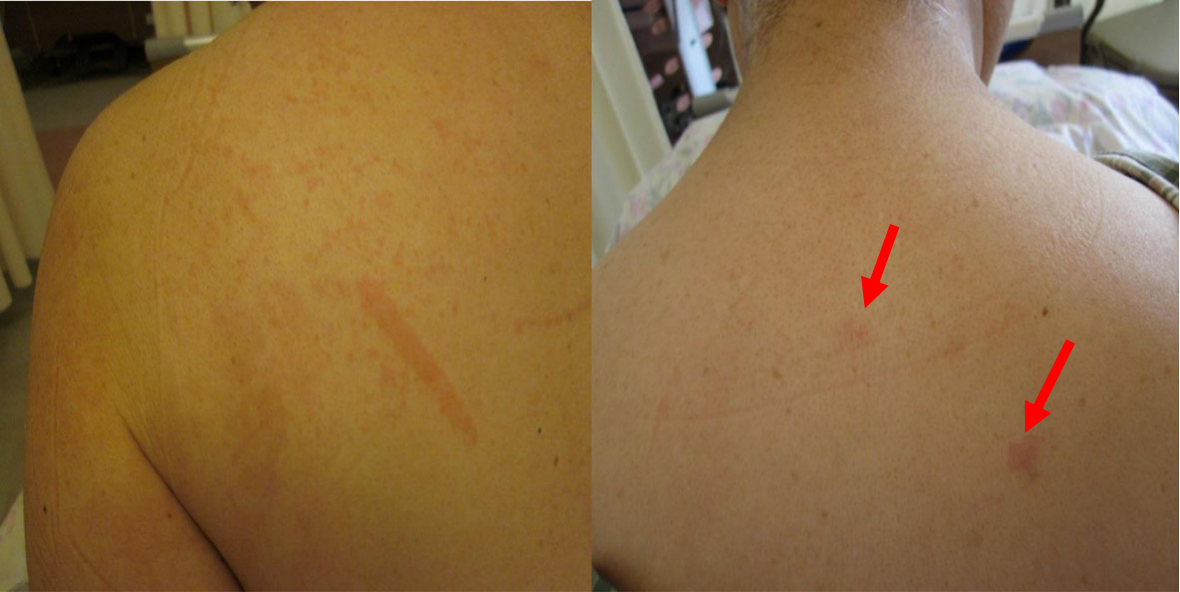
A salmon-pink papular eruption was evident on the upper back (*red arrows*)

**Fig. 2:**
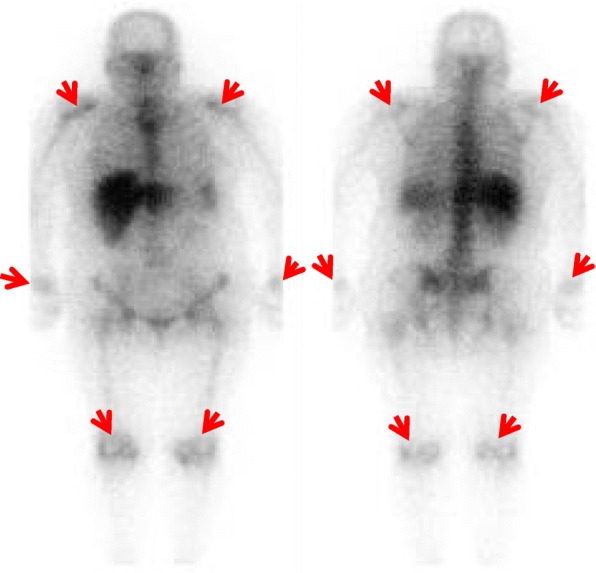
Gallium scintigraphy showed accumulation (*red arrows*)

A 66-year-old Asian woman presented to our hospital with a 2-week history of continuous high quotidian fever, pain to her right elbow and bilateral lower limbs, and erythematous rash. She was admitted for examination and treatment. Her temperature on admission was 39 °C, and it ranged from 39 to 40 °C daily. Her past medical history was negative except for celecoxib allergy. She was married; however, her bedridden husband was under care at a nursing facility due to cerebrovascular disease, and her two adult daughters lived separately. She had been engaged in cleaning work and farming until 1 month prior to her visit to our hospital. A detailed dermatological examination revealed a confluent salmon-pink papular eruption to her upper back area (Fig. 1). Further physical examination revealed mild splenomegaly and a tender right wrist. A laboratory profile revealed elevated serum ferritin levels (9692 mg/mL) but no leukocytosis. Her serum IL-18 level was markedly elevated (140,373 pg/mL); her rheumatoid factor was positive (22 IU/mL). Autoantibodies such as ANA, anti-neutrophil cytoplasmic antibody, matrix metalloproteinase-3, serologic test for hepatitis B and C, urine analysis, and 2/2 sets of blood culture were negative. Chest-abdomen computed tomography showed splenomegaly. Gallium scintigraphy showed accumulation to bilateral knees, shoulders, and wrists (Fig. 2).

**Fig. 3:**
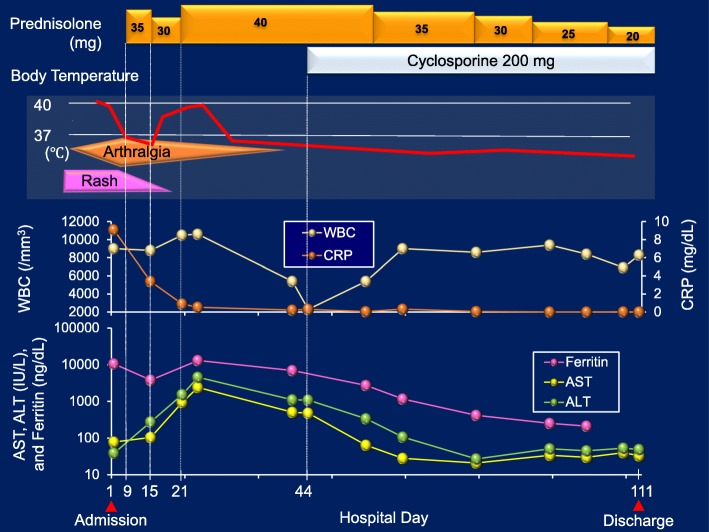
Clinical course of the patient. *ALT* alanine aminotransferase, *AST* aspartate aminotransferase, *CRP* C-reactive protein, *WBC* white blood cells

**Fig. 4:**
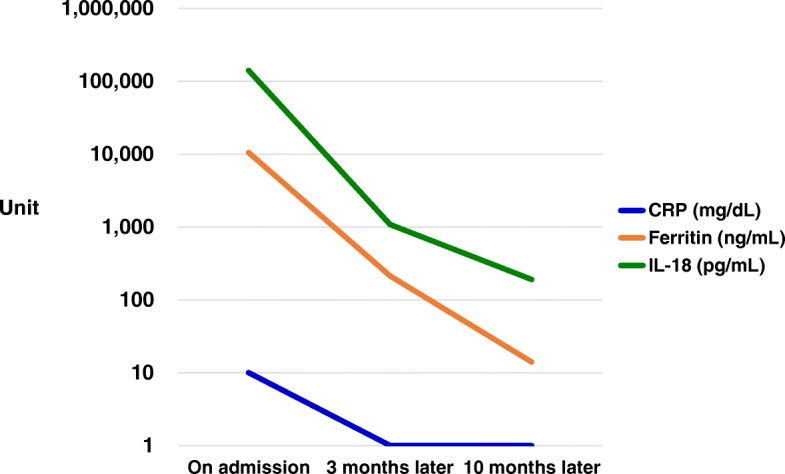
Transition of measured levels on and after admission of interleukin-18, ferritin, and C-reactive protein. *CRP* C-reactive protein, *IL-18* interleukin-18

Clinical and laboratory findings on admission were consistent with a diagnosis of AOSD based on Yamaguchi criteria [1]. Oral administration of 35 mg/day (0.5 mg/kg per day) prednisolone was started on day 9 of hospitalization; however, fever and arthralgia persisted. Therefore, prednisolone was increased to 40 mg/day and cyclosporine 200 mg/day administered orally was added on day 20 of hospitalization, which resulted in a dramatic resolution of our patient’s febrile episodes and polyarthralgia. Prednisolone was tapered 5 mg/day every 2 weeks and cyclosporine 200 mg/day was continued. Serum levels of ferritin and IL-18 on day 99 of illness were markedly decreased to 212 mg/dL and 1078 pg/mL, respectively. She was discharged on day 111 of hospitalization (Fig. 3). Cyclosporine was continued at the same dosage and prednisolone was gradually tapered. Regular follow-up examinations showed no relapse of symptoms. On day 305 of illness, her serum levels of ferritin and IL-18 were decreased to 14 mg/dL and 190 pg/mL, respectively (Fig. 4).

## Discussion and conclusions

The criteria for AOSD diagnosis proposed by Yamaguchi include high spiking fever and salmon-like evanescent rash. In addition, joint involvement and myriad nonspecific symptoms may occur [2]. AOSD diagnosis remains one of exclusion, ruling out infectious, autoimmune, or malignant conditions, and is defined by Yamaguchi classification criteria. The pivotal role of the macrophage is cell activation and release of Th1-type inflammatory cytokines [5]. It remains difficult, however, to determine predictive factors of outcome and to draw guidelines for patient management.

AOSD is rare and exhibits a bimodal age distribution with one peak incidence between ages of 15 and 25 and a second peak between ages of 36 and 46 years [6]. The frequency of AOSD in older people is unknown [7]. The immune system in older patients undergoes a functional remodeling. After menopause, the incidence of chronic inflammatory disease in women approaches or exceeds that observed in men. The immune system of aged individuals is quite different from that of a young or middle-aged adult, and these differences correlate with increased susceptibility to various infections, the reduced efficacy of some vaccinations, and a greater risk of chronic disease driven by chronic inflammation [8]. In this case our patient was greater than 65 years of age and was, therefore, likely to have some degree of immunological impairment, which could have induced AOSD. The number of older patients with AOSD is likely to increase in the future because this age group is susceptible to immunological dysfunction; therefore, awareness of this disease should be increased.

IL-18 was first described in 1999 [9]. The main source of IL-18 in humans is mononuclear cells, such as monocytes, macrophages, dendritic cells, and lymphocytes B [10]. Macrophage activation is a hallmark of AOSD, and serum from patients with AOSD discloses a dramatic increase in growth and differentiating factors for macrophages [10]. This cytokine plays effector and regulatory roles in a variety of early inflammatory responses. It is also expressed at sites of chronic inflammation, in autoimmune disease, a variety of cancers, and the context of numerous infectious diseases [11]. The dramatic effects of cytokine storms are now well documented in AOSD. The serum level of IL-18 was reported to be correlated with disease activity and ferritin serum level [6]. IL-18 could play a central role in AOSD, and systemic expression may be responsible for tissue damage in target organs such as liver, spleen, and synovial membrane [12]. This suggests an important role in resistance to intracellular pathogens that can develop inside immune cells, including macrophages [13]. Our previous report demonstrated that IL-18 reduced viral infection due to enhancement of interferon-gamma and natural killer cell activity in an animal model [14].

Higher serum IL-18 levels were related to AOSD disease activity, and a positive significant correlation was confirmed between IL-18 serum levels and disease activity [4]. Furthermore, these levels were higher than those in rheumatoid arthritis, Sjögren’s syndrome, and systemic lupus erythematosus, whose IL-18 levels were lower than 3000 pg/mL [4]. In a previous article, serum concentration of IL-18 was suggested as a diagnostic marker of AOSD; and in patients with active AOSD, it was higher than the highest value in healthy individuals and its cutoff point was 312.5 pg/mL for detection of AOSD [4]. In this case, it was greater than 100,000 pg/mL at admission.

We report the first case of AOSD in an older patient with rash typically associated with Still’s disease in which serum IL-18 level was a good biomarker of AOSD activity in both acute and convalescent phase. Serum IL-18 level could be a diagnostic marker in AOSD in older patients.

## Abbreviations

ANA: Antinuclear antibody
AOSD: Adult-onset Still’s disease
IL: Interleukin
